# Coat colour in marsupials: genetic variants at the *ASIP* locus determine grey and black fur of the brushtail possum

**DOI:** 10.1098/rsos.240806

**Published:** 2024-07-31

**Authors:** Donna M. Bond, Andrew Veale, Alana Alexander, Timothy A. Hore

**Affiliations:** ^1^ Department of Anatomy, University of Otago, Dunedin, New Zealand; ^2^ Manaaki Whenua—Landcare Research, Lincoln, New Zealand

**Keywords:** coat colour, marsupial, brushtail possum, agouti signalling protein, *ASIP*

## Abstract

The possession of fur or hair is a defining characteristic of mammals and can occur in a variety of colours and patterns. While genetic determinants of coat colour are well described in eutherian ‘placental’ mammals, the other major mammalian infraclass, marsupials, is grossly understudied. The fur of the common brushtail possum (*Trichosurus vulpecula*), an iconic native mammal found throughout Australia and introduced into Aotearoa New Zealand, possesses two main colour morphs: grey and black. To identify genetic variants associated with coat colour, we performed a genome-wide association study (GWAS) with genotype by sequencing (GBS) data. Single nucleotide variants (SNVs) on chromosome 3, close to the *agouti signalling protein* (*ASIP*) gene that controls the temporal and spatial distribution of pigments in eutherian mammals, were identified. Fine-mapping identified a C>T variant at chr3:100483705 that results in a ASIP:p.Arg115Cys missense substitution, and animals homozygous for this variant have black fur. In addition to uncovering the first genetic determinant of coat colour in a natural marsupial population, comparative analysis of *ASIP* in divergent marsupial species identified the dasyurids as having accelerated evolution, reflecting their well described diversity of coat colour and pattern.

## 1. Introduction

**Figure 1.**
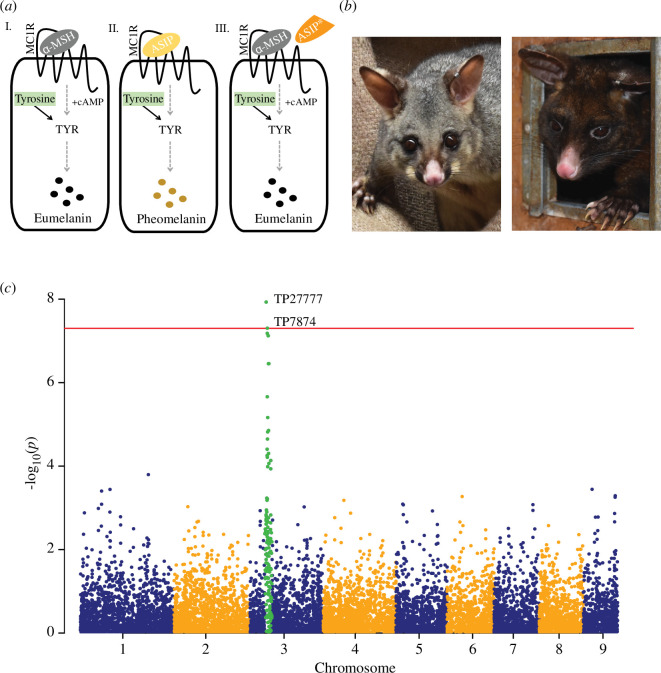
Variation in possum coat colour. (*a*) Simplified schematic representation of melanogenesis within a melanocyte (rounded square). Both pheomelanin and eumelanin (represented by mustard and black dots, respectively) are synthesized in a series of reactions (represented by grey dashed arrows), including the catalysis of tyrosine (green rectangle) by the rate-limiting enzyme tyrosinase (TYR; black arrows), into intermediate metabolites common to the (I) eumelanogenic and (II) pheomelanogenic pathways. The G-protein-coupled melanocortin 1-receptor (MC1R; represented by zig-zag line on surface of the melanocyte) is central to melanogenesis; if MC1R is bound by alpha-melanocyte stimulating hormone (α-MSH; grey oval), cAMP accumulates and eumelanin is produced (I); if agouti signalling protein (ASIP; yellow oval) is present, it will antagonize α-MSH and bind MC1R, reducing the accumulation of cAMP to promote pheomelanin production (II). Alternating bands of eumelanin (I) and pheomelanin (II) along the hair shaft give rise to a mottled grey/brown appearance that is termed agouti. Mutations that reduce the expression of *ASIP* or result in a non-functional ASIP protein (ASIP*; modified orange oval) lead to increased eumelanin production owing to the lack of α-MSH antagonism (III). (*b*) Example of a grey (left) and black (right) colour morph possum. (*c*) Manhattan plot representing the *p*-values of the entire genome-wide association study (GWAS) on a genomic scale. Single nucleotide variants (SNVs) analysed in detail (TP27777 and TP7874 at position chr3:97105906 and chr3:104107069, respectively) fall above the red horizontal line, which represents the threshold for genome-wide significance: −log_10_(5e−08).

Mammals are unique among vertebrates for nourishing their young with lactation and for the possession of fur. Mammalian fur and hair show a wide variety of colours and patterns, which can function to assist with camouflage, predator warning, thermoregulation and sexual selection [1,2]. The genetics underpinning mammalian fur colour has been extensively studied in a variety of mammals, including mice [3], humans [4], companion animals such as dogs [5,6], cats [7,8] and rabbits [9], and animals with economically important fibres such as alpacas, llamas [10] and sheep [11]. From this work, it is known that alterations in the synthesis of pigments (melanogenesis) and their transport to the skin and fibres (reviewed in [1,2]) are major determinants of coat colour variation. For example, during melanogenesis, two types of pigments can be produced: eumelanin and pheomelanin, and basic coat colour is defined by the relative proportion of the two pigments (figure 1*a*). Fur possessing only eumelanin pigment is black, while pheomelanin-only fur is yellow. Alternating bands of eumelanin and pheomelanin along the hair shaft give rise to a mottled grey/brown appearance that is termed agouti.

On a molecular level, tyrosinase (TYR) is a key rate-limiting enzyme that catalyses the conversion of tyrosine into intermediate metabolites common to the pheomelanogenic and eumelanogenic pathways; mutations in TYR lead to albinism [12]. Other significant factors central to melanogenesis include the G-protein-coupled melanocortin 1-receptor (MC1R). If MC1R is bound by alpha-melanocyte stimulating hormone (α-MSH), intracellular cAMP accumulates inside the cell, and eumelanin is produced. In contrast, if agouti signalling protein (ASIP) is present, it will antagonize α-MSH and bind MC1R, reducing intracellular cAMP accumulation and switching eumelanin production to pheomelanin [13]. Variants that reduce the expression of *ASIP*, or result in a non-functional ASIP protein, lead to increased eumelanin production. Examples of this include donkeys displaying the ‘no light points’ phenotype [14] and black-coat alpacas [15,16]. In contrast, aberrant expression of *ASIP* can lead to a loss of energy homeostasis resulting in obesity and signatures of increased pheomelanin in mice (yellow fur [3,17]) and a rare case in humans (red hair colour, pale skin and freckles [18]).

While the molecular details of fur and hair pigmentation are becoming clearer, almost all work to date has focused on eutherian, or ‘placental’ mammals. Eutherians are a mammalian clade found worldwide that reproduce with a relatively long gestation. In contrast, marsupials are found only in Australasia and the Americas, and feature development that is characterized by short gestation and relatively extended lactation [19]. While marsupials vary greatly in terms of both fur pigmentation and pattern, they are considered to follow ecogeographic and evolutionary rules and biological trends for fur trait variation like eutherian mammals [20]. Despite this, marsupials are largely unstudied from the perspective of fur genetics. The Virginia opossum (*Didelphis virginiana*) displays differential pigmentation (although more mottled pigmentation as opposed to a full black colour morph, for example) [21], and transcriptomic analysis found genes with melanocytic and immune functions to be associated with this mottled variation [22]. Targeted disruption of the TYR gene in *Monodelphis domestica* (opossum) resulted in albino offspring that carried genome-edited alleles [23], suggesting the importance of TYR in influencing skin and fibre colour in marsupials is similar to that of eutherian mammals. Nevertheless, other parts of the melanogenic pathway are yet to be explored and tested.

To address this, we studied the common brushtail possum (*Trichosurus vulpecula*), a marsupial species that naturally occurs in two pigmentation types—black and an agouti form that appears grey (figure 1*b*; electronic supplementary material, movie S1). Black possums predominate in lutruwita Tasmania (subspecies *T. vulpecula fuliginosus*) where they are locally known as timita in the palawa kani language, while grey possums are dispersed throughout the rest of their native distribution in mainland Australia (subspecies *T. vulpecula vulpecula*) [24,25]. There are many Indigenous Aboriginal names for possums in mainland Australia including walert in the Woiwurrung language in Victoria, and wilay in Wiradjuri language in New South Wales. In addition to acting as a model for other marsupials, understanding possum coat colour genetics is important considering the significance possums, and their fur, has for humans. Possums are significant for being both the most widespread marsupial in Australia [24,25], and given their ability to live and thrive in urban environments, they are also the native mammal most likely to come into human contact. For Indigenous Australians, possums and possum skins represent a cultural treasure, with possum skin cloaks used in southeastern Australia to convey stories of clan and country through decoration and etchings. Moreover, possum skin cloaks have a significant practical value historically by providing warmth [26]. The pelage of possum is soft, yet thick and woolly, and made up of many very fine, medullated fibres that trap air and add to its strong insulation properties [27]. Indeed, following the arrival of European settlers in Australia, possums were quickly exploited for clothing and rugs, becoming one of the most heavily traded furs globally by the late nineteenth century [28]. The demand for possum skins was so great that they were introduced into Aotearoa New Zealand in the 1850s to establish a new industry, with hundreds of separate introductions across the country [29]. With no natural predators, possums become an invasive pest, disrupting forest ecology through browsing plants [30,31] and opportunistic predation of native birds and invertebrates [32–34]. The majority of early importation into New Zealand was the larger, black possums from Tasmania and grey possums from Victoria [29]. Possums from these different source populations have hybridized in New Zealand; however, they are still dominated by grey and black individuals, with fewer than expected displaying intermediate fur colour, suggesting coat colour is a discontinuous trait for possums [35–37].

We recently sequenced the genome of the New Zealand brushtail possum [38], providing a platform to associate genetic variation with different traits, such as pigmentation, in this model marsupial species. We undertook genome-wide association and fine-mapping studies to identify the *ASIP* gene as the major determinant of fur colour within possums. Comparative analyses of *ASIP* in marsupials revealed novel coat colour regulation in dasyurid marsupials, a finding that we discuss alongside the potential implications of coat colour selection in hybridizing New Zealand possum populations.

## 2. Material and methods

### 2.1. Sample collection, phenotyping and metadata

Tissue samples were sourced from freshly deceased possums killed as part of pest-control programmes, and therefore did not require animal ethics oversight according to guidelines issued by the National Animal Ethics Advisory Committee [39]. The samples from Taranaki possums were initially collected as part of a landscape genomics project to assess the reinvasion rates into areas where there is an ongoing programme to eliminate the possum population [40]. Whole heads were collected and stored separately in ziplock bags and frozen. Phenotyping for coat colour was performed on the heads rather than whole bodies. Only those with clear unambiguous phenotypes were retained for genome-wide association study (GWAS; see below). For all other samples, the coat colour of the whole animal was phenotyped prior to tissue collection [38]. Tissue samples (ear or liver) were submerged in tubes filled with ethanol or RNA*later* (Invitrogen, AM7020) according to the manufacturers’ protocol. Details of sample usage in the present study are provided in table 1.

**Table 1.** Details of samples and their use in the present study.

population/sample name	number of samples	data usage	data type	references
Taranaki	215—total 187—GWAS	variant identification	GBS	Veale & Etherington [40]
Sandy assembly	1	variant identification	WGS data	Bond *et al.* [38]
Tasmanian assembly	1	variant identification	WGS data	Bond *et al.* [38]
Lawrence	47	variant screening	amplicon	Bond *et al.* [38]
Dunedin	42	variant screening	amplicon	Bond *et al.* [38]
Dunedin—skin transcriptome	6	gene expression, transcript assembly, variant screening	RNA-sequencing	this study

GBS, genotype by sequencing; GWAS, genome-wide association study; WGS, whole genome sequence.

### 2.2. DNA extractions for genotype by sequencing analysis

DNA was extracted from ear tissue (approx. 5 mm punch; approx. lentil-sized) using the DNeasy Blood and Tissue Kit (69504; QIAGEN, Hilden, Germany) on a QiaCube, with an overnight digest using proteinase K according to the manufacturer’s protocols. DNA was eluted into 200 μl of Buffer AE and stored at −20°C. The quality and quantity of the DNA were evaluated using a DeNovix DS-11 series nucleic acid spectrophotometer (DeNovix, Wilmington, DE, USA), examining the 260/230 and 260/280 ratios to determine if there was any contamination. Any sample that did not meet the criteria for purity (260/280 = 1.7−2.1, 260/230 = 1.9−2.2) or that did not have a concentration greater than 40 ng μl^−1^ was excluded and DNA extraction was repeated at least once.

### 2.3. Genotype by sequencing analysis

All DNA extractions were diluted to a uniform 50 ng μl^−1^ (with a concentration step using a SpeedVac for samples that had low concentration), and 1 μg of DNA of each sample was sent for GBS sequencing at Genomnz Animal Genomics Group (AgResearch, New Zealand). Procedures followed [41] after [42] with the following modifications: briefly, DNA was digested with PstI and MspI restriction enzymes (R0140L and R0106L: New England Biolabs, Ipswich, MA, USA)—these enzymes were chosen based on Agilent 2100 Bioanalyser (Agilent Technologies, Santa Clara, CA, USA) traces showing an even digestion pattern and no evidence of repeat sequences through the region of interest. Following ligation to barcoded adapters, the uniquely barcoded individuals were pooled into five multiplexed libraries of 94 samples. Libraries post-pooling were run through polymerase chain reaction (PCR) in multiples of four and pooled again before column clean-up, then each library was further purified and size selected (193–500 bp) using a BluePippin (2% DF Marker L; CDF2010; SAGE Science, Beverly, MA, USA). Each library was sequenced on a single lane of a single flow cell on a HiSeq2500 (Illumina, San Diego, CA, USA) using single-end reads, with 101 cycles in high-output mode (v4 chemistry). Quality checks and adapter removal followed [41]. Raw fastq files were quality checked using FastQC v. 0.10.1 (http://www.bioinformatics.babraham.ac.uk/projects/fastqc/). Barcodes and adapters were removed using cutadapt [43], then a random 15 000 reads were checked for contamination using BLAST+ against the NT database (https://ftp.ncbi.nlm.nih.gov/blast/db/), with the following settings: blastn -query - -task blastn -num_threads 2 db nt -evalue 1.0e−10 -dust '20 64 1' -max_target_seqs 1 -outfmt '7 qseqid sseqid pident evalue staxids sscinames scomnames sskingdoms stitle. The second approach to check for contamination used kmers to provide a high-level overview of the sequence composition, as described in [44]. A reference-free catalogue of single nucleotide variant (SNV) loci was then generated following [41] and the general guidelines of [45]. Via their flanking sequences, SNVs were mapped onto the recently completed possum reference genome (GCF_011100635.1) [38] using bwa mem [46] with default settings. All of the following data filtering steps were then conducted using vcftools [47]. Once sex was determined for all samples, filtered datasets were created by removing the two sex chromosomes along with any SNVs not assigned to the nine autosomes, and further filtering was conducted with the following parameters: individuals with a lower average coverage per locus than two were removed; a maximum percentage missing data per locus of 70% (--max-missing 0.3) was allowed; minimum mean depth per locus (--min-meanDP 2); maximum mean depth per locus (--max-meanDP 40); Hardy–Weinberg filtering (--hwe 0.001); minor allele count (--mac10).

### 2.4. Genome-wide association study analysis of genotype by sequencing data

After filtering, 187 individuals were retained for the genome-wide association study (GWAS), with 134 grey possums and 53 black possums. A total of 9705 SNVs were retained across the autosomes for this analysis, with a total genotyping rate of 0.724086. Plink v. 1.9 [48] was used to conduct the GWAS analyses, following the methods of [49]. The results of the GWAS were visualized using the R package fastman [50].

### 2.5. RNA extractions

Total RNA was extracted from ear punch tissue samples (approx. 50 mg), which were stored in RNA*later*, using TRIzol Reagent (15596026; Invitrogen, Waltham, MA, USA) following the manufacturer’s guidelines. Any contaminating DNA was removed from the RNA samples using the Turbo DNA-*free* kit (AM1907; Invitrogen) according to the protocol. The quality and quantity of the RNA were evaluated using a DeNovix DS-11 series nucleic acid spectrophotometer, examining the 260/230 and 260/280 ratios. The concentration of DNase-treated RNA was determined using the Qubit RNA High Sensitivity Kit (Q32852; Invitrogen) with the Qubit Fluorometer. Metadata of samples used for RNA sequencing are given in electronic supplementary material, file S1.

### 2.6. Generation of RNA-sequencing libraries

Poly(A)-enriched, strand-specific RNA-sequencing libraries were prepared using the NEBNext Ultra II Directional RNA Library Prep Kit for Illumina and NEBNext Poly(A) mRNA Magnetic Isolation Module (E7760L and E7490L; New England Biolabs) with the following modifications of the manufacturer’s protocol: the reaction volumes were reduced to half of the standard reaction volume with 250 ng of DNase-treated RNA was used as input; the NEBNext Adaptor was diluted 25-fold (0.6 μM); and the resulting libraries were amplified for 11 cycles with 0.4  μM indexed Truseq-type oligos for Illumina sequencing (electronic supplementary material, table S2). The final libraries were pooled in equimolar amounts and the pool was size selected (270–570 bp) using the BluePippin (2% DF Marker V2; BDF2010; SAGE Science). The size-selected pool was run on a NextSeq2000 with P2 100 cycle reagents (Illumina) to generate 120 bp single-end reads.

### 2.7. Analysis of RNA-sequencing libraries

Raw reads were trimmed using Trim Galore! (v. 0.6.10) to remove adapters and low-quality base calls (Phred score less than 20) (https://github.com/FelixKrueger/TrimGalore). Trimmed reads were mapped to the possum reference genome (GCF_011100635.1) using HISAT2 (v. 2.2.1) [51], with the following parameters: hisat2 -p 8 --dta --rna-strandness R. Mapped reads were visualized in IGV (v. 2.16.2) and SeqMonk (v. 1.48.1) (https://www.bioinformatics.babraham.ac.uk/projects/seqmonk/). Amplicon contamination from the opposing strand (introduced from a previous experiment using the same unique 8 bp index) was detected and removed using grep. Aligned RNA-sequencing reads were assembled using StringTie (v. 2.2.0) [52] with the following annotation-guided assembly parameters: stringtie -p 8 G --rf -A -C. The resulting StringTie-generated GTF files containing the assembled transcripts per sample were loaded into SeqMonk and visualized alongside the aligned RNA-sequencing reads. The Wiggle Pipeline within SeqMonk was then used to view the quantified density of reads mapping to the assembled transcripts. The abundance (transcripts per million, TPM) of each assembled transcript determined by StringTie was also assessed for each sample.

### 2.8. Genetic variation analysis

Whole genome sequencing analysis and SNV calling to identify heterozygous sites in both the reference assembly (Sandy) and Tasmanian assembly were described previously [38]. Mapped reads and variants sites were visualized in IGV (v. 2.16.2).

### 2.9. DNA extractions for amplicon sequencing

DNA was extracted from tissue (approx. 3  mm ear punches; equivalent to approx. half a lentil) using a modified Bio-On-Magnetic-Beads (BOMB) protocol that uses solid-phase reversible immobilization (SPRI) carboxyl-coated Sera-Mag Magnetic SpeedBeads (45152105050250, Cytiva, Buckinghamshire, UK) [53]. Tissue was lysed in 300 µl low SDS TNES buffer (100 mM Tris pH 8.0, 25 mM NaCl, 10 mM EDTA, 1% w/v SDS) with 5 µl Proteinase-K (20 mg ml^−1^) at 55°C overnight. Lysates (75 µl) were then mixed with 21 μl of ‘salty’ beads (Sera-Mag beads diluted in 5 M NaCl) before being placed on a neodymium magnetic rack until the solution was clarified (approx. 5 min). Next, 75 µl of the supernatant was transferred to a new tube containing 75 µl of ‘ethanol’ beads (Sera-Mag beads diluted in 100% ethanol), mixed by pipetting and placed at −20°C for no less than 1 h. Tubes were then placed back on the magnetic rack to clarify the solution, and the supernatant was discarded. The DNA, bound to the beads, was washed twice with 70% ethanol. Excess ethanol was removed, and beads were air-dried before resuspending the beads and eluting in nuclease-free water. DNA concentrations were measured using the Qubit DNA high sensitivity dsDNA Kit and Qubit Fluorometer. The metadata of samples used for amplicon analysis is given in electronic supplementary material, file S2.

### 2.10. Amplicon sequencing

A dual-indexing, four-primer PCR-based assay was used for amplicon sequencing, as described in [38]. Both the first and second rounds of PCR amplification were performed using Phusion High-Fidelity DNA Polymerase (M0530L; New England Biolabs) according to the manufacturer’s protocol. For the first round of amplification, approximately 10−25  ng of DNA was used in the following PCR mix: 1× HF Buffer with 1.5 mM MgCl_2_, 0.2 mM of each dNTP, 0.5 µM of each primer and 0.02 U µl^−1^ units of Phusion, with the following PCR cycling conditions: 98°C for 3 min; 27 cycles of 98°C for 10 s, 62°C for 20 s, and 72°C for 15 s; 72°C for 2 min. The PCR reaction was cleaned up and size selected using 0.9× SPRI beads diluted in standard PEG buffer (18% w/v polyethylene glycol 8000 (PEG), 1 M NaCl, 10 mM Tris (pH 8.0), 1  mM EDTA, 0.05% v/v Tween-20) [53]. The DNA was eluted in 10  µl nuclease-free water, and 2  µl was used as a template in a second round of PCR amplification (using the same PCR conditions as described above) with 0.2  µM of each second step primer (indexed Truseq-type oligos for Illumina sequencing). PCR cycling parameters for the second step PCR were 98°C for 3 min; 5 cycles of 98°C for 10 s, 62°C for 20 s and 72°C for 15 s; 72°C for 2 min. The final libraries were pooled in equimolar amounts, cleaned up and size selected using 0.9× SPRI beads diluted in standard PEG buffer and sequenced on the iSeq100 (Illumina) to generate 150  bp paired-end reads. All primers used for amplicon sequencing are given in electronic supplementary material, table S2.

### 2.11. Amplicon sequencing analysis

Sequencing reads were imported into Geneious v. 2022.2.2 and trimmed using the BBDuk plugin. Trimmed reads were aligned to an ‘*in silico*’ PCR product, which corresponds to the sequence extracted from the possum reference genome (GCF_011100635.1; chr3:100483628–100483799; see electronic supplementary material, table S2 for corresponding primer sequences). The ‘Find Variations/SNPs’ feature within Geneious was used to determine the nucleotide sequence(s) at the variant site of interest, with a minimum variant frequency of 0.2. The coverage (total number of reads), reference read count and variant read count was extracted for each amplicon and used to plot the variant frequency for each sample (electronic supplementary material, file S2).

### 2.12. Protein and coding sequence alignments

ASIP protein sequences of various mammalian species were extracted from the National Center for Biotechnology Information (NCBI) and imported into Geneious v. 2022.2.2 (http://www.geneious.com) (electronic supplementary material, file S3). The ‘Pairwise/Multiple Align’ function in Geneious was used to align the protein sequences with a MUSCLE alignment and default parameters. The coding sequences of these proteins were also extracted from NCBI and imported into Geneious v. 2022.2.2 (electronic supplementary material, file S3). To identify the *ASIP* genes in the genomes of various Dasyuromorphia species, the yellow-footed antechinus ASIP protein sequence (or domains within this sequence) was used for tblastn searches of the respective genome assemblies (electronic supplementary material, file S3). The sequences of the resulting hits were imported into Geneious v. 2022.2.2, and the open-reading frame of the identified coding exons was checked and translated into protein. The ‘Pairwise/Multiple Align’ function in Geneious was used to perform a second alignment with all protein sequences using the MUSCLE alignment with default parameters (electronic supplementary material, figure S3). The resulting alignment was used to generate a neighbour-joining tree with the Geneious Tree Builder, rooted to the eutherian mammal clade as the outgroup. Bootstrap resampling was performed 1000 times.

## 3. Results

### 3.1. Genome-wide association identifies a conserved coat colour gene in possum

In northern Taranaki in New Zealand, possums have mixed coat colour phenotypes, with no observable spatial patterns in coat colour. Of a sample of 215 possums that could be phenotyped, 67% were grey, 27% were black and 6% were brown. To identify genetic variants associated with coat colour in possum, genotyping by sequencing (GBS) data was used as input into a genome-wide association study (GWAS). The GWAS was performed on coat colour (grey versus black) retaining only those individuals where the phenotype was unambiguous (*n* = 187). After filtering, 9705 SNVs were retained, and the GWAS identified two SNVs (TP27777 and TP7874) on chromosome 3 (NC_050575.1) at position 97105906 and 104107069, respectively, with high association levels with this phenotype (figure 1*c*). Seventy-nine annotated genes reside within the 7 Mbp region between the two coat colour-associated SNVs (electronic supplementary material, table S1), including the locus encoding ASIP (chr3: 100479698 – 100483755 ; XP_036604605.1), a known determinant of coat colour in eutherian mammals [3–5,10].

### 3.2. The possum *ASIP* gene

**Figure 2.**
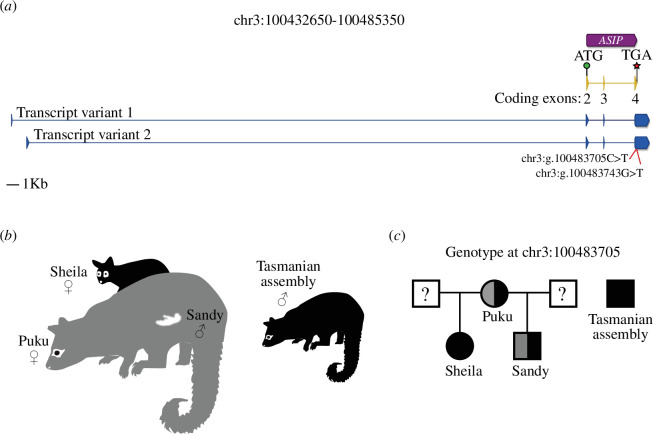
Characterization and identification of genetic variation of the *ASIP* gene in possum. (*a*) Schematic representation of the *ASIP* gene in possum (chr3:100432650–100485350 from reference genome assembly: GCF_011100635.1). The NCBI pipeline annotation of *ASIP* in possum (chr3: 100479698 – 100483755) is represented by the purple box, with the arrowhead depicting the direction of the gene. The coding sequence of the NCBI pipeline annotated *ASIP* transcript is in yellow and the assembled *ASIP* transcripts from RNA-sequencing libraries of possum skin are in blue. Exons are represented by vertical boxes (the arrowhead depicts the direction) and introns as horizontal lines. The name of each NCBI-annotated coding exon is given under each exon. The start of translation (ATG codon) is represented by a green circle and the termination codon (TGA) is represented by a red star. Genomic coordinates of the NCBI-annotated and RNA-sequencing assembled exons are presented in electronic supplementary material, file S1. The position of the two genetic variants found in exon 4 is represented by red lines. (*b*) Schematic representation of coat colour phenotype for Sandy (male pouch young; colour unknown as fur had not yet developed), Puku (Sandy’s mother, grey), Sheila (Sandy’s half-sister, black) and the Tasmanian assembly individual (black). (*c*) Pedigree showing the genotype at position chr3:100483705 for individuals presented in (*b*). The coat colours of Sandy’s and Sheila’s fathers are unknown (?).

Both the temporal and spatial distribution of *ASIP* transcripts (including the variable non-coding exon 1 upstream of the coding sequence) are known to control coat colour in different mammals, such as mice, dogs, rabbits and goats [6,54–56]. To characterize the possum *ASIP* gene, as well as its expression, we undertook RNA-sequencing from ear punch skin tissue of grey (*n* = 3) and black (*n* = 3) possums from Dunedin, New Zealand. In addition to the NCBI pipeline annotated transcript, we identified two alternative transcripts that each included an untranslated exon 1 (of variable length), separated from coding exons 2–4 by a large intron (46.02 and 44.65 kb, respectively) (figure 2*a*; electronic supplementary material, file S1). Interestingly, both transcript variants were found in skin tissue from grey and black-furred possums (electronic supplementary material, figure S1 and file S1), and no clear pattern of expression could explain coat colour. Furthermore, variation in transcript levels was highly variable between individuals of the same coat colour (electronic supplementary material, figure S1). This is consistent with transcriptomic analysis of skin pigmentation variation in the Virginia opossum, where *ASIP* expression was not found to be differentially expressed between pigmented and non-pigmented animals [22].

### 3.3. Identification of variants in the possum *ASIP* gene

As *ASIP* gene regulation did not explain linkage to coat colour, we undertook fine mapping of *ASIP* variation by screening for heterozygous sites within the coding region of *ASIP*. We first used the possum reference genome (GCF_011100635.1) called ‘Sandy’ [38]*,* as this genome was sequenced to high depth (60.7× coverage), allowing confident SNV calling. Moreover, although the reference genome was derived from a pouch-young without fur or pigmentation, his mother ‘Puku’ had grey fur and his half-sister ‘Sheila’ had black fur, implying the reference may have been heterozygous for coat-colour variants (figure 2*b*) [35,36]. In addition, we also analysed the genome of a Tasmanian possum known to have had black fur [38]. A total of 27 single nucleotide variants and one 10 bp deletion were found in the *ASIP* locus of the reference genome (table 2). Of these variants, only two resided in the coding sequence of *ASIP*, specifically exon 4. The first variant, chr3:g.100483705C>T, results in a ASIP:p.Arg115Cys missense substitution, and the second variant, chr3:g.100483743G>T, results in a ASIP:p.Arg127Arg synonymous substitution. The black coat Tasmanian possum was homozygous T/T for both variants (table 2).

**Table 2.** Genetic variants identified at the *ASIP* locus in possum.

variant location on chromosome 3 (NC_050575.1) in reference assembly (GCF_011100635.1)	variant in reference assembly (Sandy)	variant in Tasmanian assembly	position in *ASIP* gene	CDS change	amino acid change
100479279	C/T	T/T	upstream of start codon	—	—
100479343	C/T	T/T	upstream of start codon	—	—
100479397	A/G	G/G	upstream of start codon	—	—
100479446	A/G	G/G	upstream of start codon	—	—
100479465–100479475	TTCTGTTTCC/T	T/T	upstream of start codon	—	—
100479484	T/C	C/C	upstream of start codon	—	—
100479541	G/A	A/A	upstream of start codon	—	—
100479865	G/A	A/A	intron 1	—	—
100479910	G/A	A/A	intron 1	—	—
100480015	C/T	T/T	intron 1	—	—
100480036	G/A	A/A	intron 1	—	—
100480108	G/A	A/A	intron 1	—	—
100480205	C/T	T/T	intron 1	—	—
100480262	C/T	T/T	intron 1	—	—
100480416	A/T	T/T	intron 1	—	—
100480640	T/C	C/C	intron 1	—	—
100480650	G/A	A/A	intron 1	—	—
100480771	A/C	C/C	intron 1	—	—
100480810	G/C	C/C	intron 1	—	—
100480819	C/T	T/T	intron 1	—	—
100480859	G/T	T/T	intron 1	—	—
100481586	C/T	T/T	intron 2	—	—
100481593	T/G	G/G	intron 2	—	—
100481599	G/A	A/A	intron 2	—	—
100481994	C/T	T/T	intron 2	—	—
100483705	C/T	T/T	exon 4	CGC > TGC	R > C
100483743	G/T	T/T	exon 4	CGG > CGT	R > R

CDS, coding sequence.

### 3.4. Association of the identified *ASIP* mutation in possum with black coat colour

We hypothesized that the chr3:g.100483705C>T variant, when in a homozygous T/T state, is responsible for the lack of functional ASIP, and results in black coat colour within the possum. Our RNA-sequencing data from the grey and black possums supported this hypothesis: grey-furred possums expressed either just the C allele or both the C and T allele, but black-furred possums only expressed the T allele (electronic supplementary material, figure S1).

To further test this linkage of chr3:g.100483705C>T with coat colour, we established an amplicon sequencing pipeline to genotype possums with a recorded coat colour phenotype for this genetic variant. We started our analysis by genotyping Sandy’s family, and as predicted for the recorded phenotypes, Puku (Sandy’s mother) was heterozygous C/T and Sheila (Sandy’s half-sister) was homozygous T/T, consistent with their grey and black fur, respectively (figure 2*b,c*). We then expanded our analysis to include diverse populations with greater numbers.

**Figure 3.**
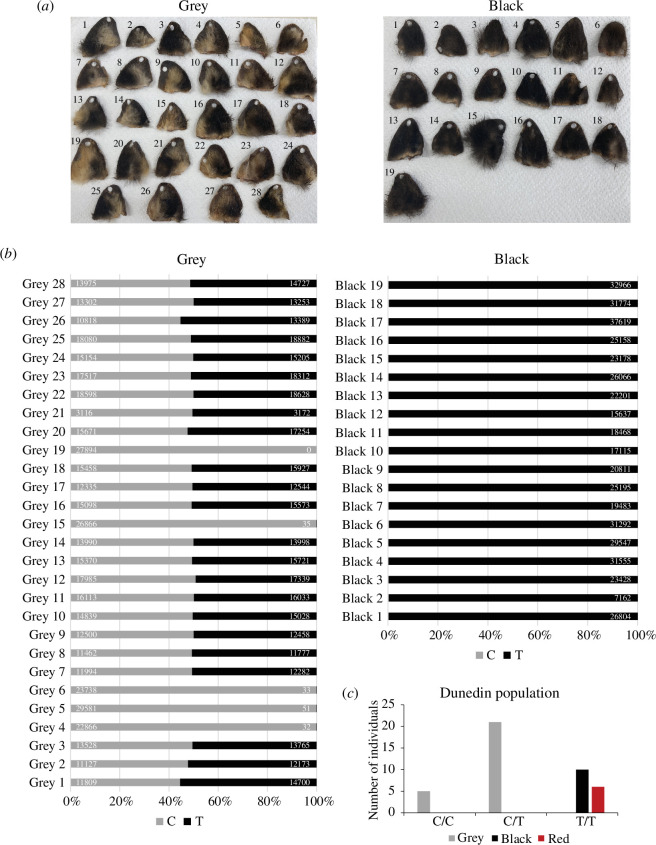
The chr3:g.100483705C>T variant is linked to black coat colour in possum. (*a*) Possum ears collected from grey (left) and black (right) animals around Lawrence (Otago, New Zealand). (*b*) Allele frequency at position chr3:100483705 in possums with grey (left) and black (right) fur (from *a*). The reference ‘C’ allele is grey, and the variant ‘T’ allele is black. Values represent the read number for the C allele (left) and the T allele (right) from the amplicon sequencing data for each sample. (*c*) The number of possums phenotyped as grey, black or red in the Dunedin population that were genotyped as homozygous C/C, heterozygous C/T or homozygous T/T at position chr3:100483705.

The first population was from Lawrence (Otago, New Zealand), where possum ears were collected for DNA and coat colour was phenotyped (figure 3*a*). There was a strong distinction in coat colour on the ears of these possums—animals phenotyped as grey had hallmarks of ‘agouti’ and golden (pheomelanin) fur, whereas the animals phenotyped as black lacked the presence of any ‘agouti’ fur. Amplicon sequencing of the variant at position chr3:100483705 revealed grey animals carried either one or two copies of the reference C allele, whereas the black animals carried two copies of the variant T allele (figure 3*b*). This result suggests the T allele, when inherited in an autosomal recessive manner, leads to the black coat colour of possums.

Consistent with the Lawrence population, black coat possums from around Dunedin (figure 3*c*) were the T/T genotype at position chr3:100483705. A small number of possums from Dunedin were phenotyped as being ‘red’ (electronic supplementary material, figure S2), and genotyping revealed these individuals were homozygous T/T at position chr3:100483705. This suggests ‘red’ fur is largely independent of the variation at *ASIP*, consistent with previous observations that deviations from ‘grey’ or ‘black’ are infrequent [35–37,57], and both colour morphs can display a rufous tinge [58], which may increase with age [37]. Although the T allele was at a higher frequency than the C allele in the Lawrence and Dunedin populations, the distribution of C/C, C/T and T/T genotypes were close to 1 : 2 : 1 and the populations did not deviate statistically from Hardy–Weinberg equilibrium (figure 3*b*,*c* and electronic supplementary material, file S2). Collectively this data unequivocally confirmed a homozygous C>T variant at position chr3:100483705, which leads to ASIP:p.Arg115Cys substitution, is the genotype of possums with black fur.

### 3.5. Comparative analysis of possum ASIP

**Figure 4.**
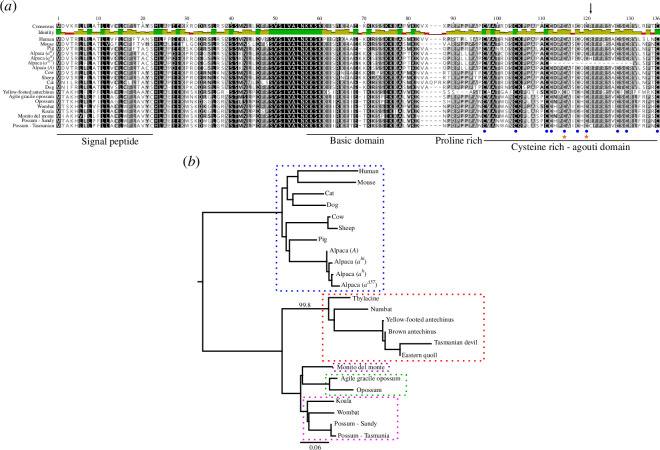
Comparative analysis of mammalian ASIP and differences in marsupials. (*a*) Protein alignment of ASIP from various eutherian and marsupial mammals. The amino acid similarity is depicted by colour: 100% similar (black); 80–100% (dark grey); 60–80% (light grey); less than 60% (white), which is summarized by the identity track: residues that are the same at the position of interest across all sequences are green, residues with 30% to under 100% identity are yellow, and sites with less than 30% identity are red. The locations of the signal peptide, basic domain, proline-rich domain and cysteine-rich agouti domain within ASIP are represented by horizontal lines. The position of the 10 cysteine residues within the agouti domain is represented by blue circles. Orange stars represent Cys residues, when mutated, give rise to black coat colours in mice (left) [59] and donkeys (right) [14]. The ASIP:p.Arg115Cys substitution identified in black fur possums (Possum—Tasmanian) is represented by the black vertical arrow. This corresponds to the same position as the ACY91947.1:p.Arg118His substitution of the alpaca *a^H^* allele [15,16]. Further details of the sequences used for the alignment are in electronic supplementary material, file S3. (*b*) Neighbour-joining phylogenetic tree reconstruction of ASIP in different marsupial orders, rooted to the eutherian mammal (Placentalia) clade as the outgroup (blue dotted box). The different marsupial orders represented include Dasyuromorphia (red dotted box); Didelphimorphia (green dotted box); Diprotodontia (pink dotted box) and Microbiotheria (purple dotted box). Sequence divergence is represented as the proportion of substitutions per site of the sequence alignment (scale bar). The protein alignment used to reconstruct the tree is in electronic supplementary material, figure S3 and details of the sequences used are in electronic supplementary material, file S3.

To investigate whether the ASIP:p.Arg115Cys substitution was found more broadly across the mammalian tree of life, we aligned the possum reference and variant ASIP amino acid sequences against ASIP sequences of 14 mammals, including six marsupial species (figure 4*a*). As expected, high conservation was observed with all Cys residues within the C-terminal agouti domain being present in species with ‘agouti’ fur. Mutation of these Cys residues gives rise to dark coat colours in mice [59] and donkeys [14]. The Cys residues pair to form five disulphide bonds, which present the highly conserved ArgPhePhe (RFF) residues of the ASIP protein to direct interaction with melanocortin receptor proteins, such as MC1R [60]. The ASIP:p.Arg115Cys substitution identified in possum is within the RFF sequence, suggesting a substitution to CysPhePhe (CFF) could affect the interaction with MC1R and thus reduce or abolish pheomelanin production (figure 1*a*). Consistent with this, homozygous variants in alpaca (*Lama pacos*; ACY91947.1:p.Arg118His) and pampas cat (*Leopardus colocola*; ASIP:p.Arg120Cys), both of which correspond to possum ASIP:p.Arg115Cys, result in animals with black fur (figure 4*a*) [15,16,61]. An Arg to Cys substitution in the critical RFF loop creates an odd number of cysteine residues, which is predicted to interfere with the folding of the disulfide-rich agouti domain [61].

The protein alignment we performed also identified a difference in the ASIP protein from yellow-footed antechinus (*Antechinus flavipes*), a member of the Dasyuromorphia (carnivorous marsupials), compared with the other mammals, including the Diprotodontia, Didelphimorphia and Microbiotheria marsupial orders. Specifically, the yellow-footed antechinus ASIP protein lacked the proline-rich domain (figure 4*a*), which, in mice, is considered to contribute to melanocortin receptor affinity and selectivity [62]. To determine if this difference was unique to the yellow-footed antechinus or also present in other dasyurids, we performed a tblastn search of the Tasmanian devil (*Sarcophilus harrisii*; hereafter ‘devil’) genome and the scaffold-level genome assemblies of the eastern quoll (*Dasyurus viverrinus*), numbat (*Myrmecobius fasciatus*), brown antechinus (*Antechinus stuartii*) and thylacine (*Thylacinus cynocephalus*) using the yellow-footed antechinus ASIP protein. Upon extraction of dasyurid *ASIP* genes, we identified open-reading frames, translated these to protein sequence *in silico* (electronic supplementary material, file S3) and performed an alignment with the ASIP proteins presented in figure 4*a* (electronic supplementary material, figure S3) to produce a phylogenetic tree to model the evolutionary relationships (figure 4*b*). Dasyurid ASIP sequences clustered together and were separated at a striking distance from other marsupial lineages (figure 4*b*; 99.8% bootstrap support). They lack the proline-rich domain and additional differences were found in the cysteine-rich agouti domain, although the conserved Cys residues and RFF motif are maintained (electronic supplementary material, figure S3). Furthermore, we were unable to identify the first coding exon in the devil *ASIP* gene (electronic supplementary material, file S3 and figure S3), suggesting a lack of ASIP protein in this species could be responsible for their striking black coat colour.

## 4. Discussion

The fur of the brushtail possum, a model marsupial, comes in two main colour forms: grey and black (figures 1*b* and 3*a*; electronic supplementary material, movie S1). Genome-wide association of SNVs and coat colour directed us to the *ASIP* gene (figure 1*c*; electronic supplementary material, table S1), a known regulator of pigmentation in vertebrates [2]. RNA-sequencing of skin tissue from grey and black possums identified two alternative transcripts (figure 2*a*; electronic supplementary material, file S1), but the genetic linkage between coat colour and the *ASIP* locus was independent of *ASIP* transcriptional regulation. Further analysis of variation within the *ASIP* gene identified a C>T variant at position chr3:100483705, which results in an ASIP:p.Arg115Cys missense substitution (table 2). The substituted amino acid is part of the highly conserved RFF motif within the cysteine-rich agouti domain, and when mutated, for example, in alpaca and the pampas cat, results in the production of eumelanin-rich black fibres [15,16,61]. Genotyping of this variant within independent possum populations, using an amplicon sequencing pipeline, identified black animals only ever carry two copies of the variant T allele indicating an autosomal recessive mode of inheritance (figures 2*c* and 3). Like the a*^H^* and *ASIP^R120C^* alleles found in black alpaca [15,16] and the black-furred pampas cat [61], respectively, we predict black-furred possums produce an ASIP protein that fails to antagonize α-MSH binding to MC1R, thus preventing the switch from eumelanin production to pheomelanin [13].

Our genetic analysis of coat colour in the possum is the first to document the genetic determinants regulating differential pigmentation in a marsupial population. Marsupials are a diverse mammalian group, and while many are convergent in form to eutherian mammals, their adaptations to the niches they occupy offer a unique perspective on mammalian diversity [19]. Our comparative analysis of ASIP from different marsupial orders identified dasyurid ASIP sequences are more divergent in amino acid sequence, indicating ASIP may have accelerated evolution in this clade; something which is reflected in their striking coat colour variation.

For example, devils lack a full-length ASIP protein (electronic supplementary material, figure S3 and file S3), suggesting a lack of antagonistic ASIP function and therefore continued α-MSH binding to MC1R resulting in eumelanin production and the black pelage of this species (figure 1*a*). Eastern quolls come in two colour morphs—either fawn or black—both with 60–80 irregularly shaped white spots on the dorsal surface of their body [63]. Hartley *et al.* [64] recently compared the *ASIP* gene of the brown eastern quoll individual sequenced to various dasyurid species, including the devil. They identified the deletion within the devil *ASIP*; however, they did not test dark fur eastern quolls in their analysis. Fur colour variation of fat-tailed dunnart (*Sminthopsis crassicaudata*), a small mouse-like dasyurid that inhabits mainland Australia, has been studied in some detail using genetic studies. When dark fat-tailed dunnarts were bred with lighter fur isomorphs, offspring inherited the light ‘agouti’ phenotype, indicating that, as in possums, agouti is dominant to dark [65]. It will be interesting to explore the molecular details of *ASIP* regulation and function in coat colour variation in the fat-tailed dunnart, eastern quolls and other sympatric dasyurids, which commonly feature variations in pattern and colour [66].

A common habitat for Australian marsupials with black fur, including possums, devils and eastern quolls, is the island of Tasmania [24,63,67]. This raises the question of what the advantage of dark fur is, and why it may have been selected for in this location. One hypothesis involves crypsis (concealment), where melanistic animals can achieve better camouflage in darker environments, irrespective of whether they are predators or prey [1]. The devil is now the apex predator of Tasmania following the extinction of the thylacine in the mid-twentieth century. Current hypotheses suggest that thylacine preyed on juvenile devils [68], and perhaps the black coat of adult devils helped avoid their detection by the former apex predator [69]. Eastern quolls typically live separate from devils and display more vigilant behaviour in the presence of their larger competitor and potential predator [70], suggesting predation risk is a strong influence on habitat selection by eastern quolls [71]. By extension, this predation risk may also select for dark colour in eastern quolls sympatric with devils.

Black coat possums in Tasmania are found predominantly in high rainfall and heavily timbered country, with minimal diurnal temperature variation [58,72,73]. Gloger’s rule, which predicts animals and birds are darker in warmer and/or more humid habitats, with humidity having a greater influence [74], could explain this relationship [58], as has been documented for American marsupials [75]. The wet forest environment of Tasmania perhaps selected for dark fur possums leading them to gradually become the dominant colour morph in this habitat [76,77]. Conversely, possum is a prey species of the non-arboreal devil [78,79], indicating a further potential driver of black fur may be improved concealment from predators. In support of this as a significant selective force, introduction of the devil to Maria Island in 2012 to establish an insurance population, led to high possum mortality [80,81].

The distribution of coat-colour morphs in New Zealand appears largely to be a consequence of importation from Tasmania or mainland Australia [29,57]. Nevertheless, selection on possums with respect to climate may be an important determinant for the distribution of colour morphs in New Zealand. Although black possums from Tasmania were (perhaps by design) disproportionately released in colder, wetter regions, such as the west coast of the South Island [29], it has been observed that small, grey, mainland Australia-type possums tend to inhabit dry, warmer areas, and are less able to colonize cold areas with high rainfall such as the Southern Alps headwaters [37]. Coat colour may not be the driver of this—Tasmanian-derived (black fur) possums are larger, meaning they will do better than grey possums in the wet, colder environment of the west coast of the South Island, where increased body mass is advantageous during abstinence from feeding during frequent high rainfall [82,83], in accordance with Bergmann’s rule [84]. Furthermore, the smaller size of mainland (grey) possums in warmer, drier conditions, such as Whanganui on the west coast of the North Island (like warmer regions of Australia), confers advantages in water balance and heat dissipation [82]. Genomic analysis of ‘hybrid zones’ in New Zealand, where possums of both Tasmanian and mainland Australian heritage have been released and are apparently interbreeding without restriction [36,38], could be used to disentangle the contributions of body size and coat colour with respect to habitat preference. Indeed, a recent possum hybrid zone study found no link between mitochondrial ancestry and coat colour [36].

In summary, we show that a missense variant in the coding sequence and not a quantitative change in expression of the *ASIP* gene is the major determinant of coat colour morphs in the brushtail possum, an iconic Australian native species. On the basis of accelerated evolution in dasyurid marsupials, we predict that *ASIP* is not just significant for possums, but rather a fulcrum by which coat colour is modulated more broadly in marsupials.

## Data Availability

The RNA-sequencing data generated in this study has been deposited to the NCBI Gene Expression Omnibus (GEO) database under accession: GSE250237. The amplicon sequencing data generated in this study have been deposited to Dryad [85]. Supplementary material is available online [86].
